# Work-Related Pain in Extrinsic Finger Extensor Musculature of Instrumentalists Is Associated with Intracellular pH Compartmentation during Exercise

**DOI:** 10.1371/journal.pone.0009091

**Published:** 2010-02-09

**Authors:** Angel Moreno-Torres, Jaume Rosset-Llobet, Jesus Pujol, Sílvia Fàbregas, Jose-Manuel Gonzalez-de-Suso

**Affiliations:** 1 Research Department, Centre Diagnòstic Pedralbes, Esplugues de Llobregat, Spain; 2 Centro de Investigación Biomédica en Red, Bioingeniería, Biomateriales y Nanomedicina (CIBER-BBN), Esplugues de Llobregat, Spain; 3 Institut de Fisiologia i Medicina de l'Art, Terrassa, Spain; 4 Centro Radiológico Computarizado (CRC) Corporació Sanitària, Institut d'Alta Tecnologia, Barcelona, Spain; 5 Centro de Investigación Biomédica en Red, Bioingeniería, Biomateriales y Nanomedicina (CIBER-BBN), Barcelona, Spain; 6 Sports Medicine Department, Real Sociedad de Fútbol SAD, Donostia-San Sebastian, Spain; Pennington Biomedical Research Center, United States of America

## Abstract

**Background:**

Although non-specific pain in the upper limb muscles of workers engaged in mild repetitive tasks is a common occupational health problem, much is unknown about the associated structural and biochemical changes. In this study, we compared the muscle energy metabolism of the extrinsic finger extensor musculature in instrumentalists suffering from work-related pain with that of healthy control instrumentalists using non-invasive phosphorus magnetic resonance spectroscopy (^31^P-MRS). We hypothesize that the affected muscles will show alterations related with an impaired energy metabolism.

**Methodology/Principal Findings:**

We studied 19 volunteer instrumentalists (11 subjects with work-related pain affecting the extrinsic finger extensor musculature and 8 healthy controls). We used ^31^P-MRS to find deviations from the expected metabolic response to exercise in phosphocreatine (PCr), inorganic phosphate (Pi), Pi/PCr ratio and intracellular pH kinetics. We observed a reduced finger extensor exercise tolerance in instrumentalists with myalgia, an intracellular pH compartmentation in the form of neutral and acid compartments, as detected by Pi peak splitting in ^31^P-MRS spectra, predominantly in myalgic muscles, and a strong association of this pattern with the condition.

**Conclusions/Significance:**

Work-related pain in the finger extrinsic extensor muscles is associated with intracellular pH compartmentation during exercise, non-invasively detectable by ^31^P-MRS and consistent with the simultaneous energy production by oxidative metabolism and glycolysis. We speculate that a deficit in energy production by oxidative pathways may exist in the affected muscles. Two possible explanations for this would be the partial and/or local reduction of blood supply and the reduction of the muscle oxidative capacity itself.

## Introduction

Work-related musculoskeletal disorders of the upper limb are common occupational health problems that cause pain, functional deficit, loss of work time and high medical costs. Among the pathologies encompassed by such a label [1], non-specific muscle disorders are still controversial two decades after the terms “repetitive strain injury” and “overuse syndrome” were coined to describe diffuse non-specific pain in the upper limb muscles of workers engaged in mild repetitive tasks [2], [3]. The main criticisms have been the lack of scientific evidence to show that subjects exposed to static and/or highly repetitive low amplitude tasks develop tissue damage, the lack of an objective test for diagnosis and the ambiguity of the terminology employed [4]. Despite such criticisms, the condition is recurrently present in epidemiological and clinical studies, and highly prevalent in some segments of the active population. Instrumentalists are particularly prone to develop the condition, which affects up to 50% of professional symphony orchestra musicians and 21% of music students [5], [6], and, when untreated, it may severely disable them.

Much of the criticism is most probably a consequence of the lack of structural and biochemical information regarding the affected muscles, since studies in humans and animal models are scarce [7]–[11]. Some studies have shown that, in addition to changes in fiber type percentage and hyperthrophy, mitochondrial abnormalities may be present in the affected muscles [8] and data in the work-related myalgic extensor carpi radialis brevis muscle have shown an increased reliance on glycolytic metabolism during exercise [11]. Such evidence would suggest that alterations in oxidative metabolism and a shift towards glycolysis may be associated with the condition. Phosphorus magnetic resonance spectroscopy (^31^P-MRS) is a safe, non-invasive and well proven technique for the rapid monitoring of intracellular changes in high energy phosphates and intracellular pH in the muscle during exercise and provides potential for the detection of energy metabolism alterations [12]. The aim of the present study, using non-invasive ^31^P-MRS, was to compare the muscle energy metabolism during exercise of the whole finger extensor musculature in instrumentalists suffering from work-related pain with that of healthy control subjects to detect potentially useful alterations for the diagnosis and understanding of the pathophysiology of the condition. We chose the finger extensor musculature on the grounds that it is the most affected in instrumentalists, especially keyboard and string players [13]. Our results showed an intracellular pH compartmentation in the form of neutral and acid compartments, as detected by Pi peak splitting in ^31^P-MRS spectra, predominantly in myalgic muscles, and a strong association of this pattern with the condition. This is consistent with the simultaneous energy production by oxidative metabolism and glycolysis in myalgic muscles, while in non-affected muscles the major contribution to energy production was oxidative. A plausible cause for such processes is a deficit in energy production by oxidative mechanisms in the affected muscles.

## Methods

### Participants

Nineteen volunteer instrumentalists were included in the study: 11 patients and 8 healthy controls. Groups were comparable for hand and forearm anthropometry, age and sex (Table 1), and none of the subjects was involved in any regular training activity involving forearm exercise. Patient criteria for inclusion were: self-reported symptoms (diffuse muscle pain, fatigue and persisting focal tenderness) in the finger extrinsic extensor musculature of a minimum 6-month duration (range 7 months to 10 years) that significantly impaired or prevented performance; confirmation of muscle pain and focal tenderness by palpation; and exclusion of other musculoskeletal, peripheral nerve or vascular pathology by clinical examination and MRI. Symptoms were bilateral in 10 patients and one patient showed affectation only in the non-dominant forearm. Inclusion criteria for controls were absence of symptoms and normal physical examination. Detailed information as to the musicians' professional history was collected in the selection visit (Table 1). Both dominant and non-dominant arms were studied for all subjects.

**Table 1:** Descriptive data of subjects included in the study.

	Controls	Patients
Subjects (n)	8	11
Sex (Males/Females)	5/3	6/5
Age (years)	22.6±3.2	23.2±5.0
Length of the dominant hand (mm)	179±13	180±10
Muscle forearm CSA (mm^2^), dominant arm	3013±793	2961±890
Muscle forearm CSA (mm^2^), non-dominant arm	2804±720*	2767±821*
Professionals	6 (75%)	7 (64%)
Total duration of instrument playing (years)	14.3±4.1	11.6±6.5
Daily practice (hours)	3.8±1.0	3.4±1.4

Values are mean ± SD. CSA; cross-sectional area. There were no significant differences between patient and control groups in either dominant or non-dominant arms.

* Significant differences: dominant vs. non-dominant arm, paired data Student-t test, p<0.001. Instrumentalists by instrument (controls/patients): Piano (3/5), Guitar (2/0), Electric guitar (1/2), Saxophone (1/1), Clarinet (1/1), Double bass (0/1), Drums (0/1).

### Experimental Protocol

Studies were carried out using a 1.5 T Signa system operating on the spectroscopy 5.8 version (Signa Advantage, General Electric Medical Systems, Milwaukee, WI, USA). Subjects were placed in the magnet in a prone position, headfirst with their arm extended and centered. ^31^P spectra were obtained with a pre-tuned elliptical transmitter and receiver surface coil (14.5×6.5 cm) centered on the hand extrinsic extensor muscle belly located by palpation and ocular inspection of the dorsal surface of the forearm during a sustained finger extension using a home-built ergometer (see below for details).

The exercise involved rhythmic finger extension-flexion (30 cycles x min^−1^, 1-second extension and 1-second flexion) performed using a home-built ergometer. The device consisted of a series of rubber bands (elasticity constant  = 38.0±5.3 N/m, n = 10; double strand) attached between fingers and wrist, over the palm side of the hand, fixed with Velcro straps that forced the fingers to close over the palm. One band was attached to the fingers' proximal phalanx and another to the middle phalanx of the index and middle fingers, and to the proximal phalanx of the thumb. An exercise based mainly on extension was required by both extrinsic and intrinsic hand muscles to overcome the elastic resistance. On the extrinsic extensor muscles, extension was produced by a concentric contraction. Finger flexion was progressive and active against the tension produced by the rubber bands resulting in an eccentric contraction. Muscle contraction was auxotonic as both the tension and length of the involved muscles changed simultaneously. The exercise was sustained until the subject was unable to continue due to either pain or exhaustion or, alternatively, following 1000 seconds with no signs of pain or fatigue. Both the ergometer resistance and the exercise were empirically designed and previously tested in healthy controls by electromyography and ^31^P-MRS (not shown) to activate primarily the finger extensor musculature (although with some minor contributions of the abductor pollicis longus and the wrist extensor musculature), generate a low-intensity aerobic exercise and induce an energy steady state.

Energy requirements in the exercise performed depend basically on rubber band resistance and the stretched length, while muscle energy production depends on recruited muscle mass. Dominant hand length, as a surrogate of exercise energy requirement, was measured on the ventral surface from the wrist basal crease to the tip of the third digit using a bone caliper with a resolution of 0.5 mm. Muscle cross-sectional area (CSA) of both dominant and non-dominant forearms was measured on axial T_1_-weighted MR images acquired on a plane orthogonal to the forearm longitudinal axis with the standard knee coil (subject in a prone position, headfirst with arm extended) and the following parameters: 420/10 msec (repetition time/echo time), two signals acquired, 16 cm field of view, 256×192 data matrix and 4-mm slice thickness with 1-mm interslice gap. The measure was performed on the image at the level of the distal part of the radial tuberosity. Muscle tissue was manually selected and the area surrounded was calculated using General Electric standard software.

### ^31^P-MRS

Spectra were recorded using a one-pulse sequence with a 180° pulse at the coil's centre. A total of 1024 data points were collected over a spectral width of 2500 Hz. After 4 dummy scans, data acquisition was performed at rest (32 scans) and during exercise in 8-scan blocks with a scan repetition time of 2 s. The total acquisition times ranged from 1.5 to 19.5 minutes. Signal quantification was performed in the time domain in 32-scan blocks for adequate signal-to-noise ratio. Advanced Magnetic Resonance fitting algorithm (AMARES) [14], as included in the jMRUI version 2.2 software package [15], was used to fit the amplitude of phosphocreatine (PCr) and inorganic phosphate (Pi) resonances. Intracellular pH was calculated from the chemical shift of Pi relative to PCr [16]. The patterns and kinetics of PCr, Pi, Pi/PCr ratio and intracellular pH were evaluated blindly to the subjects' condition to find deviations from the expected metabolic response to exercise protocol.

### Ethics

The study protocol was approved by the Institut de Fisiologia i Medicina de l'Art ethics committee. All participants gave written informed consent.

### Statistical Methods

All data are reported as mean ± SD. Statistical analyses were performed using SPSS 8.0 statistical software (SPSS Inc.1997, Chicago, IL, USA). Data in quantitative variables were normally distributed as assessed by the Shapiro-Wilk test (not shown) and parametric tests applied. Differences between paired and unpaired data were tested by the Student t-test. Repeated measures ANOVA was applied to evaluate group and possible hand dominance effects on the observed Pi/PCr ratio values. The chi-square-test with a 2×2 table design and Yates's continuity correction was applied to evaluate a possible relationship between MRS alterations and the initial diagnosis. Pearson's correlation coefficient was used to evaluate possible correlations between MRS results or exercise time and potentially relevant anthropometric and professional history variables to explain the observed findings. Differences were considered significant at p<0.05 for all analyses.

## Results

### Exercise Findings

Exercise time in patients was twofold lower than in the controls (dominant forearm: 492±361 s (range 89–1112 s) vs 817±268 s (range 328–1172 s), p = 0.047; non-dominant forearm: 410±301 s (range 92–1143) vs 893±189 s (range 605–1172), p = 0.0009). No correlation was found between exercise time and muscle forearm CSA or hand length, but we did observe a positive correlation between hand length and dominant (r = 0.62, p = 0.0048) or non-dominant (r = 0.67, p = 0.0017) forearm muscle CSA.

**Figure 1:**
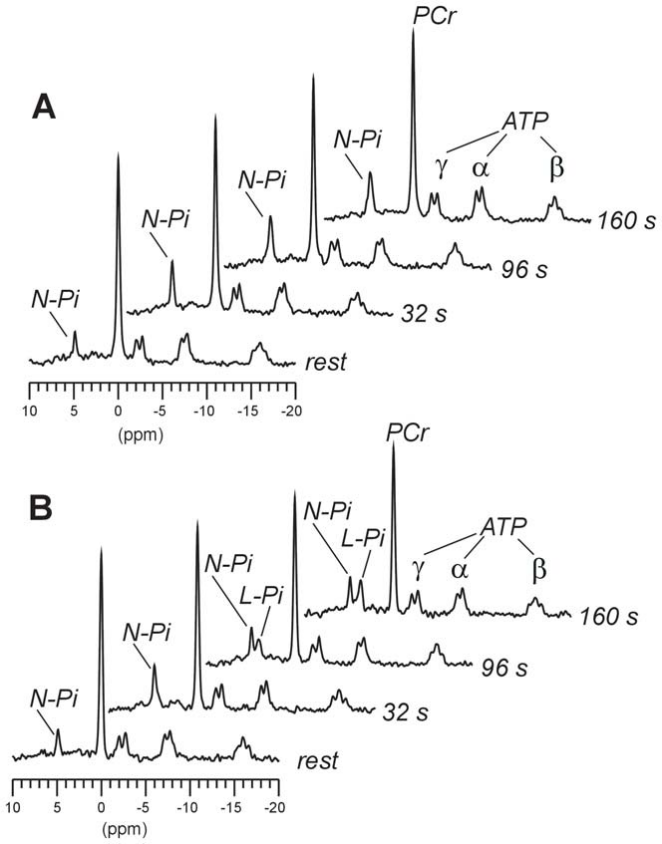
Representative ^31^P spectra of the finger extensor muscles at rest and during exercise. A, MRS pattern A (control); B, MRS pattern B (patient). Peak assignments: ATP, adenosine triphosphate; PCr, phosphocreatine; Pi, inorganic phosphate (N-Pi, neutral-pH peak; L-Pi, low-pH peak). The spectra are the sum of 32 scans (64 s) The displayed spectra times are the centre points of such time periods. Note the appearance of a second and acidic Pi peak (L-Pi) in pattern B spectra. Exercise in the chosen example of pattern A was performed for a longer time period without Pi splitting, but only the same time points as in pattern B example are shown for comparison purposes.

**Figure 2:**
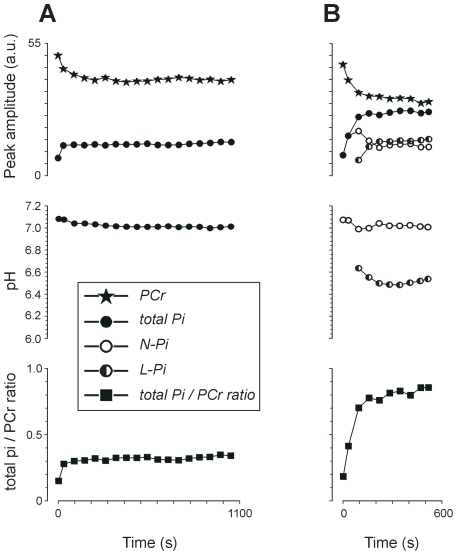
Representative time courses of metabolite raw amplitudes, intracellular pHs, and total Pi/PCr ratios during exercise. A, MRS pattern A; B, MRS pattern B; PCr, phosphocreatine; Pi, inorganic phosphate (N-Pi, neutral-pH peak; L-Pi, low-pH peak). Data were obtained from 64 s spectra and the displayed symbol times are the centre point of the time periods.

During the exercise, PCr decreased and Pi increased in extensor muscle spectra and two different Pi patterns were observed (Figures 1,2). In pattern A (16 forearms) we detected only one Pi peak, which, following an initial amplitude increase, reached a plateau (both in amplitude and pH, the latter within the neutral range) that maintained until the exercise was complete. By contrast, in pattern B, shortly after the onset of exercise (97±31 s determined at the acquisition resolution of 16 s, range 56–136 s, n = 19 forearms) another Pi peak with an acidic pH (6.54±0.17) appeared from the former and neutral Pi peak and was visible to the end of the exercise (final pH = 6.46±0.13). The neutral peak reached a plateau and remained constant as in pattern A. The low-pH Pi peak showed a fairly constant amplitude (n = 13 forearms) or a peak increase throughout the exercise (n = 6 forearms). In three forearms, from two subjects (patients), the performed exercise was too short (<136 s) to discard the possibility of splitting and could not be assigned to either Pi pattern. Results from these forearms were therefore excluded from the subsequent analysis. The total Pi/PCr ratio increased during the exercise and reached a plateau in all cases except those pattern-B forearms in which the low-pH Pi peak increased throughout the exercise. In such cases, the total Pi/PCr values also increased throughout with no plateauing.

### Association between MRS Patterns and Subject Groups

Pattern A was mainly present in controls and pattern B in patients. Pattern B, however, was also detected in three controls unilaterally and pattern A in two patients, bilaterally in one, with unilateral myalgia, and unilaterally in the other (Table 2). On a forearm basis, a significant association between myalgia and pattern B was found (χ^2^  = 15.1; p = 0.0001) with up to 89% of myalgic forearms showing pattern B compared with only 17.6% of the non-myalgic forearms showing pattern B (Table 3). No correlations were found between MRS patterns and hand length, muscle CSA or musicians' professional history.

**Table 2:** Occurrence of Pi peak splitting patterns in the studied population.

Pattern	Affectation	Controls		Patients	
		Subjects	Forearms	Subjects	Forearms
A	unilateral	3*	3 (nd)	1*	1 (d)
	bilateral	5	10	1†	2
B	unilateral	3	3 (d)	2	2 (d, nd)
	bilateral	---	---	7	14
other ‡	Unilateral	---	---	1*	1 (nd) §
	Bilateral	---	---	1	2||

* Subjects also showing unilateral pattern B.

† Clinically the affected forearm was only the non-dominant one.

‡ Exercise too short to discard the possibility of splitting (<136 s).

§ Exercise time was 92 s.

|| Exercise times in dominant and non-dominant forearm were 89 s and 105 s, respectively. d, dominant forearm; nd, non-dominant forearm.

**Table 3:** Distribution table for myalgia in finger extensor muscles vs. MRS pattern detection on a forearm basis.

	Myalgia	
MRS pattern	No	Yes
A	14	2
B	3	16

Dependence between myalgia in finger extensor muscles and MRS pattern was tested with the chi-square test. χ^2^ (1, 0.0001)  = 15.1.

ANOVA of the total Pi/PCr ratio at plateau and exercise end-point in the above-mentioned six cases with no plateau did not show any significant main effect of hand dominance, but rather a significant effect of group assignment to pattern A or B (F(1,31) = 28.5, p<0.001)). Pooling data from dominant and non-dominant forearms, approximately twofold higher values were observed in forearms showing pattern B compared with pattern A (0.67±0.23, n = 19, vs 0.34±0.10, n = 16; p<0.001). Exercise total Pi/PCr ratio values of pattern A forearms showed inverse correlations with muscle cross-sectional area (r = −0.82, p<0.001) and hand length (r = −0.54, p = 0.017). Pattern B forearms did not show such correlations, but rather an inverse correlation with exercise time (r = −0.58, p = 0.009).

## Discussion

This study provided evidence for a reduced finger extensor exercise tolerance in instrumentalists with work-related myalgia and an association between such a condition and altered MRS pattern in finger extensor musculature during the performance of a constant load low level exercise. There was a muscle pH compartmentation mostly in the myalgic musculature (89% of the study forearms with clinical symptoms) in the form of neutral and acid compartments as detected by Pi splitting. Such an MRS pattern was also detected in 37.5% of the control musicians (17.6% of the study forearms with no clinical symptoms). This agrees well with the described prevalence of 37% for normal instrumentalists with “overuse” symptoms causing no functional limitations or any perception of a physical problem that might lead to medical consultation [17]. It is likely that the control musicians with MRS alterations in the present study belong to the group of normal musicians with inconsequential symptoms from the aforementioned study. Interestingly, two of the three controls with MRS alterations in which follow-up was possible were diagnosed with the condition, six months after the study was completed, in the forearms where the MRS alteration had been found.

pH compartmentation has been previously reported in non-pathological human muscles rich in glycolytic fibers when the oxidative metabolism fails to meet energy requirements, i.e. during intense exercise or in reduced blood flow situations [18]–[23]. It has been demonstrated by means of depolarizing and nondepolarizing neuromuscular blocking agents [19], [20] that the observed pH compartmentation in human skeletal muscle reflected the metabolic differences between oxidative and glycolitic fibers. In this context, the observed muscle Pi splitting/ pH compartmentation may be an indicator of the relative occurrence of the two major muscle fiber types and/or reflect differences in recruitment patterns of fibers and/or muscle groups. In our study, although the extensor muscles were rich in glycolytic fibers [24], due to the design of the exercise performed, the expected muscle response was mainly oxidative with only one neutral pH compartment of constant kinetics (PCr, Pi, PCr/Pi and pH) as seen mostly in non-affected muscles. Consequently, it is likely that the observed pathological response primarily reflects an early recruitment of glycolytic fibers in the studied whole musculature or the glycolytic contribution of specific muscle/muscles. A plausible cause for such processes is a deficit in energy production by oxidative mechanisms in the affected muscles. Data in literature suggest evidence of a reduction in the muscle oxidative capacity itself and an oxygen supply limitation that may be involved in such a deficit. Studies in myalgic muscles showed evidence of mitochondrial abnormalities in oxidative fibers: Moth-eaten fibers, core lesions, etc. [25], and two possible major mechanisms of blood supply limitation have been proposed: the failure of the radial artery to vasodilate with exercise and chronic compartment syndrome [26], [27]. A classical compartment syndrome or a global reduction of blood supply is unlikely in our study due to the presence of a constant and neutral pH Pi peak during the exercise, which would indicate that such oxidative fibers/muscles were active and working at a constant level with no noticeable lack of perfusion. Instead, a partial and/or local reduction of blood supply, as suggested by some studies [28], is more consistent with such a finding. Moreover, some of the above-mentioned mitochondrial abnormalities can also be produced by localized ischemia [29]. Interestingly, localized damage of oxidative motor units and localized blood supply reduction are two of the injury mechanisms suggested as underlying upper extremity muscle disorders during sustained and low-intensity work [30].

Most of the present MRS findings during exercise in pH-compartmented extensor muscles compared to non-compartmented ones (higher total Pi/PCr ratio values, the inverse correlation between total Pi/PCr ratio and exercise time and no correlation with muscle CSA), and the reduced exercise tolerance may be interpreted in terms of an additional contribution by glycolytic metabolism. Pi/PCr ratio values are higher in glycolytic fibers during exercise as PCr content is more hydrolyzed, resulting in higher Pi production [31]. The inverse correlation between total Pi/PCr ratio and forearm muscle CSA in non-compartmented muscles is a consequence of the existing correlation between Pi/PCr and energy production in a fully aerobic situation [32], and also due to the fact that energy production is per unit of active muscle. The lack of such correlation in pH-compartmented muscles is consistent with the additional contribution of the glycolytic metabolism and its effect on the Pi/PCr ratio, and the inverse correlation with exercise time most probably reflects the well-known fact that muscles relying mainly on oxidative metabolism are able to perform exercise longer and reach smaller Pi/PCr ratio values for a fixed workload than those with significant contributions of glycolytic metabolism. Intracellular acidosis and inorganic phosphate accumulation are accepted causative factors in fatigue through several physiological and biochemical mechanisms [33] and proton accumulation has been associated with muscle pain. It is therefore possible that, while a portion of the fibers/muscles maintain their force output and display an oxidative metabolism, other exhibit dramatic changes with glycolysis, fatigue and pain.

### Limitations

Due to the MRS setup used, the acquired signal is a mixture from the muscles comprised in an elliptical half-volume with a minor radius of 3.25 cm, and it is therefore impossible to discriminate whether the metabolic alteration found involves fibers in the whole musculature observed or merely in specific muscles. Indeed, a recent study focusing on the myalgic extensor carpi radialis brevis showed an increased reliance on glycolytic metabolism during exercise in the affected muscles [11]. Further research to discriminate whether the observed metabolic alteration affects the whole musculature or specific muscles would be recommendable. However, due to the small cross-sectional area of the studied muscles, higher magnetic field strengths and the use of chemical shift imaging techniques would be required to improve the spatial resolution [34]. The combination in such high-field studies with spatially resolved NIRS techniques may help to determine whether a local limitation exists in oxygen or blood supply in the affected muscles identified by MRS. Also, although the discussion of the results is predominantly based on the evidence that intracellular acidosis during exercise in human skeletal muscle mainly results from increased anaerobic glycolysis, our study can neither discard nor demonstrate a possible contribution of other mechanisms to the generation of acidosis in the acidic compartment found therein, such as proton efflux and buffering, which are key factors in intracellular pH control.

In conclusion, our results provided evidence of an association between work-related myalgia and intracellular pH compartmentation in the form of neutral and acid compartments in the finger extensor musculature of instrumentalists during the performance of a constant load low level aerobic exercise. Such compartmentation is consistent with the simultaneous energy production by oxidative metabolism and glycolysis involving fibers or specific muscles. The activation of glycolysis is interpreted as abnormal and most probably due to a negative imbalance between the energy production by oxidative pathways and the energy required to perform the exercise. As for the pathophysiological mechanisms leading to such an imbalance, although a partial and/or local reduction of blood supply is likely, a reduction in the muscle oxidative capacity itself or a combination of both cannot be discarded. Although further work will be required to determine the position of the metabolic alterations found in the pathophysiologic chain of events and their possible extrapolation to other muscles and the population at large, the associations found would strongly suggest potential diagnostic value and provide a good starting point for understanding the pathophysiology of the condition and translate the results into the treatment scenario. Suggestions for immediate research include discrimination as to whether the observed metabolic alteration affects the whole extensor musculature or specific muscles on the basis of high magnetic fields and chemical shift imaging techniques, and the combination of such studies with spatially resolved NIRS techniques to determine whether a local limitation exists in oxygen or blood supply in the affected muscles identified by MRS.
